# Evaluation of optical power integrity in toric and multifocal intraocular lenses

**DOI:** 10.1186/s12886-025-04380-6

**Published:** 2025-10-13

**Authors:** Hyeongju Han, Jae Woong Koh

**Affiliations:** 1 Department of Medicine, College of Medicine, Chosun University, Gwangju, Republic of Korea; 2 Department of Ophthalmology, College of Medicine, Chosun University, Gwangju, Republic of Korea

**Keywords:** Intraocular lens, Optical bench system, Optical power

## Abstract

**Background:**

Implanting an intraocular lens (IOL) with an appropriate dioptric power is critical for achieving high-quality visual outcomes after cataract surgery. Discrepancies between labeled and actual dioptric power may affect IOLs selection and compromise refractive accuracy. This study aimed to evaluate the agreement between labeled and actual dioptric powers of commercially available toric and multifocal IOLs.

**Methods:**

Spherical and cylindrical powers of toric IOLs, and spherical and additive powers of multifocal IOLs, were measured via an optical bench system based on the magnification method. Measured values were assessed for compliance with International Organization for Standardization (ISO) 11979-2 tolerance limits via two one-sided t-tests(TOST).

**Results:**

All three toric IOLs satisfied the ISO tolerance criteria for both spherical and cylindrical power (*p* < 0.05), with errors of 0.208 ± 0.170 D and 0.172 ± 0.206 D, respectively. The average absolute deviation across all toric measurements was 0.137 D. All four multifocal IOLs also met the ISO tolerance criteria for spherical and addition power (*p* < 0.05), with errors of 0.069 ± 0.041 D and 0.045 ± 0.044 D, respectively. The average absolute deviation in the multifocal group was 0.057 D.

**Conclusions:**

These results confirm that both toric and multifocal IOLs comply with ISO standards for labeling accuracy. However, the greater variability observed in toric lenses highlights the importance of rotational alignment and precise manufacturing, which are critical for successful astigmatic correction.

## Introduction

Cataract remains one of the most prevalent causes of visual impairment globally [1]. Surgical treatment involves extraction of the opacified human crystalline lens and implantation of an intraocular lens (IOL) within the capsular bag [2]. Accurate selection and implantation of an IOL with the correct dioptric power is essential for achieving optimal postoperative refractive outcomes [3–5]. Even minor discrepancies between the intended and achieved refractive outcomes can result in residual refractive errors, which may compromise uncorrected visual acuity, reduce patient satisfaction, and negatively impact quality of life [6, 7].

One important source of such errors is the discrepancy between the labeled power of an IOL and its actual optical power [8–10]. Norrby et al. [11] reported that this variability increases with higher dioptric power and is strongly influenced by differences in measurement methods and equipment. These findings led to the establishment of ISO 11979-2, which defines acceptable tolerance limits for labeled IOL powers [12]. Kenneth et al. [13] further validated these concerns by measuring commercially available monofocal IOLs using a confocal laser system. They observed measurable differences between labeled and actual power, especially in high-power IOLs, although all values remained within the ISO tolerance limits. These studies underline the need for consistent and standardized verification of IOL labeling accuracy.

Despite these efforts, most evaluations have focused on monofocal IOLs [11, 13]. However, recent clinical practice has increasingly used toric and multifocal IOLs to address more complex visual needs [14]. Toric IOLs are designed to correct preexisting corneal astigmatism and require precise rotational alignment for optimal effectiveness [15]. Multifocal IOLs provide simultaneous focus at multiple distances, allowing patients to maintain comfortable vision without a spectacle [16]. Both designs introduce additional optical complexity, which may affect the measurement reliability and power accuracy. This raises concerns about whether the labeling precision confirmed in monofocal IOLs also applies to more advanced models.

While previous studies have predominantly focused on monofocal IOLs, toric and multifocal IOLs present additional optical and structural complexities that may influence labeling accuracy. Given their increasing adoption in clinical settings, it is crucial to evaluate whether these advanced IOLs also comply with ISO standards. This study aims to fill this gap by assessing the dioptric power accuracy of both toric and multifocal IOLs using an ISO-compliant optical bench system and statistically validating their labeling accuracy through equivalence testing.

## Methods

### Intraocular lenses

Four intraocular lens (IOL) models from the TECNIS platform (Johnson & Johnson Vision) were evaluated in this study: ZXT375, ZCT150, ZLB00, and ZKB00. Among these four models, ZXT375, ZLB00, and ZKB00 were each evaluated at two different labeled powers, resulting in a total of seven individual IOLs analyzed in this study (Table 1). All the IOLs were fabricated from hydrophobic acrylic material with a refractive index of 1.47 and shared a one-piece design featuring a 6.0 mm optic diameter and a 13.0 mm overall length.

**Table 1:** Main characteristics of seven IOLs

Manufacturer	Brand name (Model)	Material	Spherical Diopter (D)	Cylindrical Diopter (D)	Addition Diopter (D)	Refractive index
Abbott Medical Optics (now Johnson & Johnson Vision)	TECNIS Symfony Toric(ZXT375)	Hydrophobic acrylic	20	3.75	-	1.47
Abbott Medical Optics (now Johnson & Johnson Vision)	TECNIS Symfony Toric(ZXT375)	Hydrophobic acrylic	21.5	3.75	-	1.47
Abbott Medical Optics (now Johnson & Johnson Vision)	TECNIS Toric(ZCT150)	Hydrophobic acrylic	22.5	1.5	-	1.47
Johnson & Johnson Vision	TECNIS Multifocal(ZLB00)	Hydrophobic acrylic	16	-	3.25	1.47
Johnson & Johnson Vision	TECNIS Multifocal(ZLB00)	Hydrophobic acrylic	17	-	3.25	1.47
Johnson & Johnson Vision	TECNIS Multifocal(ZKB00)	Hydrophobic acrylic	17	-	2.75	1.47
Johnson & Johnson Vision	TECNIS Multifocal(ZKB00)	Hydrophobic acrylic	17.5	-	2.75	1.47

ZXT375 and ZCT150 are toric IOLs designed to correct preexisting corneal astigmatism. The ZXT375, part of the TECNIS Symfony Toric Extended Range of Vision IOLs line, incorporates an echelette diffractive structure to extend the range of vision by elongating the focal zone. This design enhances intermediate visual performance while minimizing contrast sensitivity loss and dysphotopsia. It features a labeled cylindrical power of + 3.75 D at the IOL plane. In contrast, the ZCT150 is a conventional monofocal toric IOL labeled with + 1.50 D cylinder at the IOL plane, suitable for correcting mild regular astigmatism.

ZLB00 and ZKB00 are multifocal diffractive IOLs intended to provide simultaneous vision at multiple focal points. The ZLB00 is labeled with a near addition of + 3.25 D at the IOL plane, optimized for enhanced near visual acuity. ZKB00, with + 2.75 D near addition, is designed to provide improved intermediate vision while maintaining functional near focus. Both multifocal IOLs share the same platform and material composition but differ in their diffractive profiles and optical addition powers, resulting in distinct visual performance characteristics.

### Optical bench

The power of each IOL was measured via the OptiSpheric^®^ IOL R&D system (Trioptics GmbH) based on the magnification method and in compliance with ISO 11979-2 Annex A.4 [12]. This method determines the power of the IOLs by measuring the magnification of the entire optical bench including the IOL under test. The measurement is performed using a Light source that emits monochromatic Light with a wavelength of 546.1 nm [17]. The light is formed by a collimator that projects the image of the double slit to infinity. This parallel beam passes through the IOL immersed in a medium (in this study, PBS) and produces an image of the object in the focal plane of the lens under test. This image is collected by a microscope and focused onto a high-resolution charge-coupled device (CCD) camera. The camera automatically adjusts its position to find the best focus point until the image of the double slit is clearly detected on the monitor. At the optimal focus position, the size of the object image is precisely determined with subpixel accuracy by the CCD camera. The effective focal length (EFL) is calculated from the measured magnification, and the refractive power is determined as the reciprocal of the EFL. The system provides measurement accuracies ranging from ± 0.1% to ± 0.3% over the refractive power range.

### Experimental setup

All optical measurements were performed according to ISO 11979-2 for IOL power validation. Prior to measurement, each IOL was fully hydrated in phosphate-buffered saline (PBS; pH 7.4; Welgene Inc.) at room temperature (20 °C) for at least 3 h to stabilize the refractive index. Bennett et al. [18] measured the power change of the IOL with temperature change and reported that the optical power of the IOL reached equilibrium after 80 min. Accordingly, the IOL was placed in PBS maintained at 35 ± 2 °C for at least 90 min to achieve thermal equilibrium. This thermal conditioning step ensured that the refractive index of the PBS (approximately 1.336) was similar to the refractive index of aqueous humor, providing physiologically relevant measurement conditions.

The IOLs were measured via OptiSpheric^®^ IOL R&D platform. Based on the manufacturer’s guidelines and the technician’s recommendation, an aperture size of 5.0 mm was selected for the toric IOLs to sufficiently cover the cylindrical optical region, while a 3.0 mm aperture was used for the multifocal IOLs.

Each IOL was measured independently six times. Before each test, the lens was completely removed from the PBS solution and repositioned on the optical axis. This process introduces intentional positioning variability to account for alignment-related deviations, thereby improving the reliability and repeatability of the results.

### ISO tolerance criteria

The assessment of measurement accuracy was based on the tolerance Limits defined in ISO 11979−2. These limits vary depending on the labeled spherical, cylindrical, and addition power, as well as the spherical equivalent (SE) of each lens. Table 2 summarizes the specific criteria used to determine equivalence in this study.

**Table 2:** ISO 11979-2 tolerance limits for spherical(S), cylindrical(C), and addition(A) powers

Power Type	Labeled Power Range (D)	Tolerance limits (D) for SE* < 25D	Tolerance limits (D) for SE ≥ 25D
Spherical Power	0 < |S| ≤ 15	± 0.30	
	15 < |S| ≤ 25	± 0.40	
	25 < |S| ≤ 30	± 0.50	
	|S| >30	± 1.00	
Cylindrical Power	0 < C ≤ 2.5	± 0.30	± 0.40
	2.5 < C ≤ 4.5	± 0.40	± 0.40
	4.5 < C	± 0.50	± 0.50
Addition Power	0 < A ≤ 2.5	± 0.30	± 0.40
	2.5 < A ≤ 4.5	± 0.40	± 0.40
	4.5 < A	± 0.50	± 0.50

*SE (spherical equivalent) is calculated as SE = S + C/2​, where S is the spherical power and C is the cylindrical power, and is used to determine the applicable tolerance range

### Statistical analysis

All analyses were performed in Python (v3.11, Google Colaboratory) using pandas and SciPy [19, 20]. Equivalence between measured and labeled refractive powers was evaluated with the Two. Equivalence testing was conducted using the two one-sided tests (TOST) approach [23]. One-Sided Tests (TOST), implemented as two one-sided t-tests under the assumption of normality [22]. TOST was run in Python using the statsmodels package. Normality was verified with the Shapiro–Wilk test [21]. Equivalence margins were set to the ISO 11979-2 tolerance limits as presented in this manuscript, which constitute the regulatory acceptance criteria for labeling accuracy. We report both lower and upper one-sided p-values from TOST because the procedure tests simultaneously against the lower and the upper equivalence bounds.Statistical significance was set at *p* ≤ 0.05.

## Results

### Evaluation of toric IOLs

**Fig. 1:**
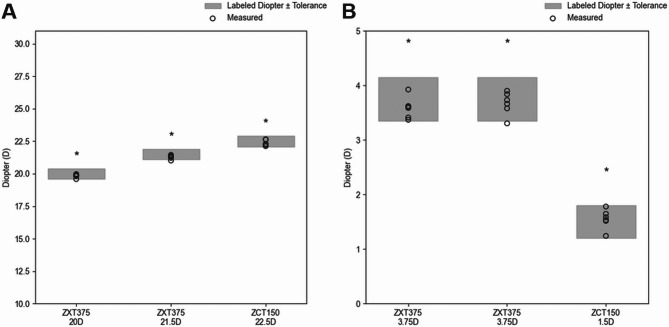
Equivalence analysis of labeled and measured dioptric powers for toric IOLs. The plots show the measured spherical powers (**a**) and cylindrical powers (**b**) for three toric IOL models: ZXT375 (20.0 D and 21.5 D) and ZCT150 (22.5 D). Gray regions indicate the labeled power ± tolerance limits. Black circles represent individual measured values (*n* = 6 for each group). Asterisks (*) denotes statistical equivalence to the labeled power was confirmed via TOST(*p* ≤ 0.05 for both bounds)

Three toric IOL models ZXT375 (20 D and 21.5 D) and ZCT150 (22.5 D) were evaluated in this study (Table 3). For each lens, both the spherical and cylindrical powers were measured, with each measurement repeated six times (*n* = 6). The mean error for spherical power was 0.208 ± 0.170 D, and that for cylindrical power was 0.172 ± 0.206 D. The average absolute deviation across all the components was 0.137 D, and the mean standard deviation was 0.169 D, indicating good measurement repeatability and labeling precision. Equivalence to the labeled power was assessed via TOST, with all tested values showing statistical equivalence (*p* < 0.05). Although a few individual measurements exceeded the ISO tolerance limits, the overall error remained within acceptable statistical bounds. Figure 1 illustrates the distribution of the measured powers relative to the labeled values and ISO tolerance limits. These findings confirm that the evaluated toric IOLs demonstrate sufficient labeling accuracy and meet international standards for clinical use.

**Table 3:** Labeled and measured powers with TOST equivalence results for three toric IOLs (n = 6)

Model	Power Type	Labeled Power (D)	Tolerance limit (D)	Average	Standard Deviation	*p*-value (upper)	*p*-value (lower)
ZXT375 (20D)	Spherical	20.0	0.4	19.838	0.121	< 0.001	0.003
	Cylindrical	3.75	0.4	3.588	0.180	< 0.001	0.016
ZXT375 (21.5D)	Spherical	21.5	0.4	21.284	0.139	< 0.001	0.016
	Cylindrical	3.75	0.4	3.670	0.195	0.001	0.007
ZCT150	Spherical	22.5	0.4	22.349	0.213	0.001	0.024
	Cylindrical	1.5	0.3	1.550	0.163	0.009	0.002

### Evaluation of multifocal IOLs

Compared to toric IOLs, multifocal IOLs showed notably lower deviation and measurement variability, likely due to their simpler spherical structure and reduced orientation sensitivity.

**Fig. 2:**
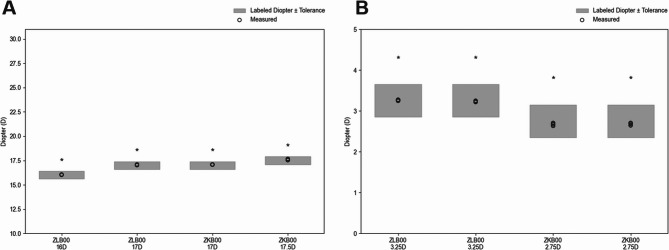
Equivalence analysis of labeled and measured dioptric powers for multifocal IOLs. The plots show the measured spherical powers (**a)** and addition powers (**b**) for four multifocal IOL models: ZLB00 (16.0 D and 17.5 D) and ZKB00 (17.0 D and 17.5D). Gray shaded regions indicate the labeled power ± tolerance limits. Black circles represent individual measured values (*n* = 6 for each group). Asterisks (*) denotes statistical equivalence to the labeled power was confirmed via TOST(*p* ≤ 0.05 for both bounds)

The multifocal IOLs ZLB00 and ZKB00 were assessed at two labeled powers each, resulting in four total lens conditions (Table 4). For each lens, both the spherical and addition powers were evaluated through six repeated measurements (*n* = 6). As shown in Fig. 2, all individual measurements fell within the ISO tolerance Limits. The mean error for spherical power was 0.069 ± 0.041 D, and that for addition power was 0.045 ± 0.044 D. The overall average absolute deviation across all components was 0.057 D, with a mean standard deviation of 0.023 D, indicating high measurement consistency and labeling accuracy. Statistical equivalence to the labeled powers was confirmed in all cases via TOST, with both p-values for the upper and lower bounds being < 0.001. These results demonstrate that the evaluated multifocal IOLs not only satisfy ISO tolerance limits but also exhibit excellent manufacturing precision and optical stability under bench-based testing.

**Table 4:** Labeled and measured powers with TOST equivalence results for four multifocal IOLs (*n* = 6)

Model	Power Type	Labeled Power (D)	Tolerance limit (D)	Average	Standard Deviation	*p*-value (upper)	*p*-value (lower)
ZLB00 (16D)	Spherical	16.0	0.4	16.038	0.012	< 0.001	< 0.001
	Addition	3.25	0.4	3.263	0.011	< 0.001	< 0.001
ZLB00 (17D)	Spherical	17.0	0.4	17.055	0.027	< 0.001	< 0.001
	Addition	3.25	0.4	3.232	0.011	< 0.001	< 0.001
ZKB00 (17D)	Spherical	17.0	0.4	17.081	0.010	< 0.001	< 0.001
	Addition	2.75	0.4	2.671	0.029	< 0.001	< 0.001
ZKB00 (17.5D)	Spherical	17.5	0.4	17.602	0.055	< 0.001	< 0.001
	Addition	2.75	0.4	2.682	0.025	< 0.001	< 0.001

## Discussion

The present study evaluated the dioptric power accuracy of three toric and four multifocal IOLs using an ISO-standard optical bench platform. Labeling accuracy was assessed according to ISO 11979-2 tolerance limits and validated through equivalence testing with the two one-sided t-test (TOST) procedure. Overall, all tested lenses satisfied ISO criteria, thereby confirming that commercially available toric and multifocal IOLs generally demonstrate reliable labeling accuracy.

For toric IOLs, the mean absolute deviation was 0.137 D with a standard deviation of 0.169 D, indicating slightly greater variability compared to multifocal designs. This finding is consistent with previous reports showing that toric IOLs are more sensitive to alignment and measurement conditions due to their cylindrical component. Clinically, even a 10° misalignment can reduce the corrective effect by one-third, underscoring the importance of both accurate labeling and rotational stability for optimal outcomes [24, 25]. Our findings extend earlier optical bench investigations on monofocal and toric lenses [11, 13], confirming that labeling accuracy is generally preserved but variability is higher in toric designs.

In contrast, multifocal IOLs showed remarkably low measurement variability, with a mean error of 0.057 D and a standard deviation of 0.023 D. All individual measurements fell well within ISO tolerance limits. These results are in agreement with prior optical quality studies on diffractive multifocal lenses, which also demonstrated consistent reproducibility and manufacturing precision [16, 17]. The reduced variability of multifocal lenses in our study may be attributable to their symmetric diffractive profiles and lower dependence on rotational orientation. The difference in measurement variability between toric and multifocal IOLs may be attributed to differences in optical design and orientation sensitivity. Toric IOLs, by virtue of their cylindrical correction, are inherently more sensitive to alignment. Even minimal deviations in rotational axis can introduce significant power errors [24, 25]. Accordingly, variability arising from structural complexity or measurement procedures should not be overlooked, as it may translate into clinically meaningful refractive outcomes. Strengths of the present study include the use of an ISO-compliant optical bench system (OptiSpheric^®^ IOL R&D), repeated independent measurements to enhance reproducibility, and inclusion of toric and multifocal designs that are less frequently studied than monofocal models. These features increase the robustness and clinical relevance of our findings. Limitations must also be acknowledged. ISO 11979−2 defines IOL power in paraxial terms, whereas all optical bench systems—including the one used here—determine the effective focal length at best focus through finite apertures. This paraxial-best focus discrepancy is a universal limitation of bench testing. In addition, only a limited number of IOL models from a single platform were evaluated, restricting generalizability across manufacturers. Finally, this study focused exclusively on dioptric accuracy without including complementary optical quality metrics such as modulation transfer function (MTF) or Strehl ratio, which would provide additional insight into visual performance. In summary, both toric and multifocal IOLs tested in this study complied with ISO 11979-2 tolerance limits for labeling accuracy, though toric IOLs exhibited greater variability. These findings highlight the importance of ISO-compliant testing, careful clinical consideration of rotational stability in toric IOLs, and the need for future investigations that incorporate additional optical quality parameters for a more comprehensive evaluation.

## Conclusions

All the tested IOLs showed statistically validated agreement with their labeled powers. The multifocal IOLs demonstrated high measurement consistency, whereas the toric IOLs exhibited slightly greater variability, likely due to their orientation-sensitive design. Future studies should incorporate additional optical quality metrics, such as modulation transfer function(MTF) and Strehl ratio, to provide a more comprehensive evaluation of IOL performance and to enhance the clinical relevance of in vitro measurements.

## Data Availability

The datasets used and/or analyzed during the current study are available from the corresponding author on reasonable request.

## Abbreviations

IOL: Intraocular lens
ISO: International organization for standardization
CCD: Charge-coupled device
PBS: Phosphate-buffered saline
EFL: Effective focal length
SE: Spherical equivalent
TOST: Two one-sided t-tests
EDOF: Extended depth of focus
